# Isolation and Characterization of Novel *Escherichia coli* O157:H7 Phage SPEC13 as a Therapeutic Agent for *E. coli* Infections In Vitro and In Vivo

**DOI:** 10.3390/biomedicines12092036

**Published:** 2024-09-06

**Authors:** Md. Sharifull Islam, Jie Fan, Md Suzauddula, Ishatur Nime, Fan Pan

**Affiliations:** 1 Center for Cancer Immunology, Institute of Biomedicine and Biotechnology, Shenzhen Institute of Advanced Technology, Chinese Academy of Sciences, Shenzhen 518055, China; smbgb101287@yahoo.com; 2 Department of Pathology, School of Basic Medicine, Henan University of Science and Technology, 263 Kaiyuan Avenue, Luoyang 471023, China; fanjie277185479@gmail.com; 3 College of Agriculture and Natural Resources, National Chung Hsing University, Taichung 40227, Taiwan; mdsuzauddula@gmail.com; 4 Key Laboratory of Environment Correlative Dietology, College of Food Science and Technology, Huazhong Agricultural University, Wuhan 430070, China; smbgb101287@gmail.com

**Keywords:** *E. coli* O157:H7, phage, isolation, characterization, SPEC13, in vivo, phage therapy

## Abstract

*Escherichia coli* O157:H7 is a recognized food-borne pathogen causing severe food poisoning at low doses. Bacteriophages (phages) are FDA-approved for use in food and are suggested as natural preservatives against specific pathogens. A novel phage must be identified and studied to develop a new natural preservative or antimicrobial agent against *E. coli* O157:H7. The phage SPEC13 displayed broad host range and was classified within the *Ackermannviridae* family based on its observed characteristics by a TEM and genome analysis. In 10 min, this phage achieves a remarkable 93% adsorption rate with the host. Its latency period then lasts about 20 min, after which it bursts, releasing an average of 139 ± 3 PFU/cell. It exhibited robustness within a pH range of 4 to 12, indicating resilience under diverse environmental circumstances. Furthermore, SPEC13 demonstrated stability at an ambient temperature up to 60 °C. A whole genome and phylogenetics analysis revealed that SPEC13 is a novel identified phage, lacking a lysogenic life cycle, antibiotic resistance genes, or genes associated with virulence, thereby presenting a promising biological agent for therapeutic application. Animal studies showed that SPEC13 effectively controlled the growth of harmful bacteria, resulting in a significant improvement in colon health, marked by reduced swelling (edema) and tissue damage (mucosal injury). The introduction of SPEC13 resulted in a substantial decrease in quantities of *E. coli* O157:H7, reducing the bacterial load to approximately 5 log CFU/g of feces. In conclusion, SPEC13 emerges as a promising inclusion in the array of phage therapy, offering a targeted and efficient approach for addressing bacterial infections.

## 1. Introduction

*E. coli* O157:H7 is a significant serotype of *E. coli* known for its pathogenicity and ability to cause severe illnesses in both humans and animals [1]. This strain is particularly concerning due to its low infectious dose and high virulence, making infections potentially life-threatening, especially for vulnerable populations [2]. *E. coli* O157:H7 is associated with a variety of clinical presentations in individuals, encompassing self-restricted watery diarrhea to more critical conditions such as thrombotic thrombocytopenic purpura and hemolytic uremic syndrome [3]. A prominent characteristic of *E. coli* O157:H7 is its capacity to adhere to epithelial cells and generate attaching and effacing (AE) lesions, which are prevalent attributes of other enteropathogenic *E. coli* strains as well [4]. Additionally, *E. coli* O157:H7 has been found to colonize the recto-anal junction of cattle, with some animals acting as super shedders of the bacterium, contributing to its transmission and prevalence [5]. Research has shown that the rumen serves as a crucial site for the long-term carriage of *E. coli* O157:H7, acting as a potential reservoir for fecal shedding of the bacterium [6].

The *E. coli* O157:H7 presents a significant financial and health burden globally, with vulnerable populations, such as different age groups and low-income countries, facing particular challenges. This pathogen is a major foodborne threat, causing a substantial number of infections, hospitalizations, and deaths each year [7]. Within the borders of the United States exclusively, *E. coli* O157:H7 is accountable for numerous instances of illness on an annual basis, resulting in hospitalizations and fatalities [7]. The pathogen’s minimal infectious threshold and heightened virulence contribute to the severity and life-threatening nature of the infections, particularly impacting young children, the elderly, and individuals with compromised immune systems [8]. Studies have shown that direct contact with cattle, their environment, or unknown sources can also lead to human infections, highlighting the importance of understanding and managing the transmission pathways of this pathogen [9]. In low-income countries, where food safety measures may be less stringent, the risk of *E. coli* O157:H7 contamination in animal products like milk and cheese is a significant concern [10,11].

Preventing *E. coli* O157:H7 infections poses significant challenges due to various factors. One key obstacle is the ability of this pathogen to cause re-infection, as previous exposure does not confer immunity against subsequent infections by the same strain [12]. The co-evolution of phages specific to *E. coli* O157:H7 highlights the adaptability of the pathogen, making it challenging to control through phage-based interventions [13]. Moreover, the antimicrobial resistance patterns observed in *E. coli* O157:H7 strains isolated from cattle in certain regions, such as West Java, Indonesia, raise concerns about the effectiveness of traditional treatment approaches [14]. Furthermore, the presence of attaching and effacing lesions in the terminal rectum of cattle infected with *E. coli* O157:H7 highlights the challenges in eradicating the pathogen from livestock populations, as these lesions facilitate colonization and shedding of the bacterium [15]. The intermittent colonization of conventionally reared lambs by *E. coli* O157:H7 at the terminal rectum mucosa further complicates control efforts in animal populations [16]. However, the ability of *E. coli* O157:H7 to cause reinfection, the identification of super shedders, antimicrobial resistance patterns, contamination of food products, zoonotic transmission, and the presence of colonization sites in livestock pose significant barriers to preventing diseases caused by this pathogen.

The rise of antibiotic-resistant bacterial strains has spurred the search for alternative treatments, with phage therapy gaining attention as a potential solution for multi-drug-resistant pathogenic bacteria [17]. Phages are highly effective against pathogenic infectious bacterial strains due to their specificity, ability to infect and kill bacteria, and potential for coevolution with bacterial pathogens [18]. The capacity of phages to manage enteropathogenic *E. coli* infections across diverse animal models, such as mice, calves, piglets, and lambs, has been documented [19]. This ability to target specific bacterial strains makes phages a promising alternative to conventional antibiotics, especially in the face of increasing drug-resistant bacterial strains [20]. On top of that, the use of antibiotics in treating infections caused by *E. coli* that produce Shiga toxin is highly controversial due to the risk of inducing prophages because the Shiga toxins can create severe complications such as bloody diarrhea, hemorrhagic colitis, and hemolytic uremic syndrome [21]. A study demonstrated that antibiotics, among other stress factors, can effectively induce Stx prophages, leading to the production of Shiga toxins. The release of Shiga toxins is a critical factor in the pathogenesis of Shiga-toxin-producing *E. coli* infections, causing damage to the intestinal lining and resulting in bloody diarrhea [22]. The specificity of phages allows for precise targeting of pathogenic bacteria while preserving beneficial microbiota. Moreover, phages can play a role in modulating the host immune response. For instance, phages have been shown to induce the production of specific mucosal antibodies in response to *E. coli* O157:H7 colonization, contributing to the innate immune response against the pathogen [15]. This interaction between phages and the host immune system highlights the potential of phage therapy in enhancing host defenses against bacterial infections.

Novel virulent phages could offer a cost-effective mechanism for managing *E. coli* O157:H7 in environmental contexts without compromising food safety, public health, or the viability of another indigenous microbiota. The aim of this study is to identify and characterize a novel *E. coli* O157:H7 phage, designated as SPEC13, in order to assess its efficacy as a treatment for *E. coli* infections in in vitro and murine models. This study also involved the isolation and purification of phage SPEC13, followed by detailed characterization of its genomic structure, morphology, and lytic activity against *E. coli* O157:H7 strains. Furthermore, this study assessed the efficacy of phage SPEC13 in reducing *E. coli* O157:H7 populations in in vivo mouse models, focusing on its biocontrol capabilities and therapeutic potential. By elucidating the properties and effectiveness of phage SPEC13, this study aims to contribute valuable insights into the development of phage-based therapies for combating *E. coli* infections in animal models, with potential implications for future therapeutic applications in clinical settings.

## 2. Materials and Methods

### 2.1. Bacteria Strains and Growth Conditions

*E. coli* strain ATCC 43890 served as the host for phage isolation in the study. A sum of 44 distinct bacterial strains, comprising 35 *E. coli* variants and 9 other bacterial types, were utilized for assessing both the phage lytic spectrum and host range. Preservation of these strains was carried out at −80 °C with 20% (*v*/*v*) glycerol. Preceding the experimental procedures, all bacterial strains underwent cultivation on a Luria–Bertani agar (LA), utilizing the streak plate technique at 37 °C. Prior to experimental procedures, all bacterial strains were streaked onto Luria–Bertani (LB) agar plates and incubated at 37 °C. Isolated colonies were then cultured overnight on a MacConkey’s (MC) and Eosin Methylene Blue (EMB) agar at 37 °C to ensure purity. The determination of phage titers employed the double layer agar plates method, where an upper layer containing 0.70% agar was utilized in conjunction with a second layer containing 1.50% agar.

### 2.2. Phage Isolation and Purification

Sixteen putative distinct phages were obtained from samples of sewage collected at the Shenzhen wastewater treatment plant in China using methodologies previously outlined [23]. The approach to isolating phages was adapted from established protocols [24]. A quantity of 5 mL of sample was passed through a 0.22 µm filter alone with 10.0 mL of LB broth and 2.50 mL of the specific bacterial strain of interest, and subsequently incubated at a temperature of 37 °C overnight. Following the period of incubation, the mixtures were collected, subjected to centrifugation at a rate of 10,000× *g* for a duration of 10 min at a temperature of 4 °C, and the resulting supernatants were filtered using a 0.22 μm pore size filter. Then, the solution, referred to as phage lysate, was obtained and utilized in spot assays for phage identification. Any phage plaques that were identified were purified by isolating individual plaques and subsequently propagating them at least six times to guarantee the phage isolate’s purity. The procedure for phage propagation had been previously documented [25]. To generate high-titer phage stocks, the phage stock (100 µL) (approximately 9 log PFU/mL) was combined with the host bacteria culture (100 µL) (ATCC 43890) in 50 mL of LB broth and incubated for 12 h at 37 °C with agitation at 160 rpm. Then, the culture was centrifuged for 10 min with 7000× *g*. Then, a supernatant was passed through a disposable syringe (sterile) filter (0.22 µm). For further experimentation, the phages were stored at 4 °C.

### 2.3. Lytic Spectrum Determination by Spot Test

The ability of the phages to cause lysis in different bacterial strains was evaluated through the spot test technique. In this method, 5 µL of phage lysates were spread on lawns formed with the 44 bacterial strains previously mentioned, which were mixed with the LB medium containing 0.7% agar on the LA plates. Subsequently, the phage lysates (5 μL) were applied to the lawns and the plates were incubated at 37 °C overnight. After the incubation period, any bacterial lawn exhibiting a lysis zone was considered susceptible to the phages.

### 2.4. Determination of Host Range by Efficiency of Plating (EOP)

The phage SPEC13, which exhibited the broadest lytic range in spot assays and the highest lysis efficiency in the killing assay, underwent further analysis regarding its efficiency of plating (EOP). An assessment of the host range was conducted through the EOP procedure, utilizing the adapted methodologies outlined in the literature [26]. The phage was subjected to consecutive dilution steps and underwent triplicate evaluation on a vulnerable bacterial host. The bacterial strains were cultured until they reached the exponential growth phase at a temperature of 37 °C. Subsequent to the incubation period, a mixture of 100 µL of bacterial culture and 100 µL of diluted phage lysate was prepared for double-layer plate experiments. Subsequent to this step, the Petri dishes were incubated for a duration of 8 h at a temperature of 37 °C. The quantity of plaques was counted, and the relative efficiency of plating (EOP) was subsequently calculated (by dividing the average plaque-forming units on the test bacteria by the average plaque-forming units on the host bacteria). The average EOP was categorized into distinct groups: an EOP of 0.5 to 1.0 denoted high efficiency; an EOP of 0.1 to < 0.5 signified moderate efficiency; values falling between 0.001 to < 0.1 indicated low efficiency; and values < 0.001 implied inefficiency.

### 2.5. Transmission Electron Microscopy (TEM) of SPEC13

Before conducting transmission electron microscopy, a concentrated solution of phage with a high titer (>10 log PFU/mL) was mixed with ammonium acetate and subjected to negative staining using 2% phosphotungstic acid (PTA) [27]. Subsequently, the phage particles were affixed to a copper grid and visualized under a Philips CM12 transmission electron microscope (Tecnai G2 Spirit; FEI, Hillsboro, OR, USA), followed by analysis using Digital Micrograph Demo 3.9.1 software (Gatan, Pleasanton, CA, USA).

### 2.6. Absorption Kinetics and One Step Growth Curve of Phage SPEC13

Adsorption kinetics experiments were performed using *E. coli* O157:H7 ATCC 43890 in the logarithmic growth phase (A_600nm_= 0.6). Phage SPEC13 was added at a multiplicity of infection (MOI) of 0.001. To evaluate SPEC13 adsorption, the infection was halted at 0, 5, 10, 15, and 20 min [28]. The samples were then centrifuged at 7000× *g* for 5 min, and the supernatant was filtered through a 0.22 μm filter. Unbound phage titers were quantified using the double-layer agar plate method. The adsorption percentage was calculated relative to the initial phage titer (no bacteria control). One-step growth experiments were conducted in accordance with the procedures outlined by Islam et al. (2021), albeit with a specific modification [29]. The fundamental approach of the experiment entailed the combination of 500 μL of phage suspension (4 log PFU/mL) with an equal volume of bacterial culture (7 log CFU/mL) to achieve a defined multiplicity of infection (MOI) of 0.001. Subsequent to this step, the resultant mixture underwent incubation at 37 °C for a duration of 10 min to facilitate phage adsorption, after which centrifugation at 7000× *g* for 5 min was carried out to sediment the bacterial cells. Following centrifugation, the supernatant was carefully decanted to remove any unattached phages, leaving behind the bacterial pellet adorned with bound phages, which was then subjected to two rounds of washing with LB medium. Post-washing, the pellet underwent resuspension in 10 mL of LB and was subjected to further incubation at 37 °C with agitation at 160 rpm for a duration of 120 min. Next, the quantification of phage titers was performed utilizing the double-layered agar plate technique to construct a comprehensive one-step growth curve for the experimental system.

### 2.7. Temperature and pH Tolerance of Phage SPEC13

To evaluate the thermal resilience, a phage suspension of 500 μL with a concentration of 9 log PFU/mL underwent exposure to a range of temperatures between 30 and 80 °C. After a 60-min period of incubation, the samples were collected for quantification following 30 and 60 min of incubation. The determination of the phage stock quantity was carried out through the utilization of the double-layer agar method. Within the examination of pH endurance, 100 μL of a phage suspension containing 9 log PFU/mL was individually introduced into 900 μL of an LB medium modified to pH levels from 2 to 13 using NaOH or HCl. Subsequently, the amalgamation was subjected to an incubation period at 37 °C for 60 min before phage titration took place.

### 2.8. Phage SPEC13 Genome Sequencing, Annotation and Comparison 

The genomic DNA of the phage was isolated and purified based on a previously published study [30]. Utilizing the HiSeq platform (Illumina, San Diego, CA, USA), the genome underwent sequencing through the paired-end library where the read length was 150 bp. Subsequent assembly of the sequences was carried out using N-ewbler (v3.0) which resulted in a singular contig. The identification of potential coding DNA sequences (CDSs) was accomplished using the CLC genomics workbench [31]. The prediction of coding genes and tRNA, along with their annotation, was facilitated by the Rapid Annotation Subsystem Technology (RAST), followed by functional annotation of CDSs through a search against the nr protein database using BLASTP. To measure the present antimicrobial-resistant genes in the phage, ResFinder-3.1 database (CGE, https://cge.cbs.dtu.dk/services/KmerFinder/, accessed on 14 October 2023) was employed. The identification of virulent factors within the phage was carried out by utilizing the VFDB database (http://www.mgc.ac.cn/VFs/main.htm, accessed on 18 October 2023). The generation of the genome map was accomplished through the utilization of the BRIG.jar, 0.95-dist software.

### 2.9. Phage Infection Assay through Challenge Testing

For the phage-infection experiment, a culture of *E. coli* ATCC 43890 grown overnight was thinned with the LB medium to a concentration of 8 log CFU/mL. Then, phage SPEC13 was introduced at a concentration of 9 log PFU/mL (MOI of 10), followed by an incubation period at 37 °C without agitation for 6 h. Control cultures without phage SPEC13 were prepared (MOI = 0). The sampling of 100 μL was conducted hourly up to 12 h, and the reduction in the cell number of viable bacteria was assessed through plating the samples on the LB agar plates.

An alternative approach involved diluting an overnight culture of *E. coli* ATCC 43890 with the LB medium to achieve a concentration of 8 log CFU/mL. Subsequently, the addition of phage SPEC13 at diverse MOIs of 0.1, 1.0, 10, and 100 was performed, followed by an incubation period at 37 °C, lasting 6 h. Control cultures without SPEC13 addition were included (MOI = 0), and a negative control using LB media only was included. The bacterial proliferation was observed at hourly intervals by measuring absorbance (A_600nm_) over a total duration of 12 h.

### 2.10. In Vivo Investigations

Male-only pathogen-free mice (C57BL/6 background strain, eight weeks old) were purchased from GemPharmatech Company (Guangzhou, China) and housed within a specific pathogen-free facility of Shenzhen Institute of Advanced Technology. The conduction of all murine experiments was duly sanctioned by the IACUC of the SIAT-CAS and executed by an authorized individual holding license number SIAT-IACUC-230612-SLG-PF-A2286. The mice were housed in a conventional polycarbonate enclosure (5 mice per cage) measuring 29 cm × 19 cm × 13 cm, furnished with sterilized bedding materials such as aspen shavings or corn cobs. The enclosures underwent weekly sanitation and bedding replacement to uphold optimal hygiene standards. The housing facility was consistently regulated at a 25 ± 2 °C temperature and 50 ± 5% relative humidity. A 12 h light/dark cycle was enforced, aligning with natural diurnal rhythms, with lights activated from 7:00 a.m. to 7:00 p.m. The mice were granted unrestricted access to a standardized diet (specific pathogen-free) and water.

The culture of *E. coli* ATCC 43890 took place in LB broth until reaching an absorbance (A_600nm_) of 0.7. Subsequently, the cells underwent centrifugation for 10 min at 4500× *g*, followed by a meticulous resuspension of the pellet in cold water at 4 °C. The cells were subjected to two washes with cold 10% glycerol, concluding with the resuspension of the pellet in 1 mL of cold glycerol and preservation at −80 °C as competent cells. Electroporation of 100 μL of cells with 0.5 ng of PCDNA3.1 was carried out under the following settings: 25 F, 2.5 kV, and 200. Subsequent to an hour of incubation in LB broth at 37 °C, the cells were plated on an LA plate supplemented with 100 µg/mL of ampicillin. Following a 12 h growth period at 37 °C in an LB with 100 µg/mL of ampicillin, the ampicillin-resistant *E. coli* strain was diluted 1:20 in fresh medium and sub-cultured for 4 h under mild aeration. The bacteria were twice washed in ice-cold PBS and subsequently suspended in cold PBS (10^9^ CFU/mL).

Three days before the initiation of experiments, the mice were divided into three groups (control, *n* = 5; disease, *n* = 5 and treatment, *n* = 5); then, oral administration of ampicillin (20 mg) in 200 µL of saline via gavage was conducted to all groups. On the third day after, the disease and treatment group were fasted for 4 h, before being orally infected with 10^8^ CFU of *E. coli* in 0.2 mL of 0.1M NaHCO_3_. Phage SPEC13 (10^9^ PFU) in 0.2 mL of 0.1M NaHCO3 was orally administered to the treatment group mice after the 3rd, 6th and 9th day of *E. coli* infusion. All phages for in vivo experiments were purified with Detoxi-Gel endotoxin removal gel (Detoxi-GelTM; Pierce Biotechnology, Rockford, IL, USA) according to the manufacturer’s protocol. Throughout the course of the experiment, each mouse was weighed individually at the same time each day, between 9:00 a.m. and 10:00 a.m.

To determine the bacterial load in mice feces, fresh fecal pellets were collected daily under sterile conditions. Each mouse was gently placed in a clean, sterile holding container for 10–15 min to encourage defecation. Fresh fecal pellets were immediately collected using sterile forceps and placed into pre-labeled sterile collection tubes. Ten-fold dilutions of resuspended fecal samples (0.1 g each) were plated on LA plates containing 100 µg/mL of ampicillin. The optimal dilution factor was determined through preliminary experiments to ensure accurate and reproducible colony counts. The bacterial colonies were counted to determine the CFU/g of feces. On the 12th day of the experiment, the mice were euthanized through exposure to ether. The entire colon was excised and measured for length in centimeters. To standardize the procedure, we consistently identified the cecum as the starting point for colon measurement and used a sterile ruler to measure the length from the cecum to the rectum. The portion of colons were subsequently preserved in a solution of 4% formalin to facilitate future histological analysis [32]. Fixed tissues were processed through graded ethanol solutions, embedded in paraffin, sectioned at 4–5 μm thickness, and placed on glass slides. The sections were stained with Hematoxylin and Eosin (H&E) for general histopathology, with additional stains such as Alcian Blue or Masson’s Trichrome used as needed. The stained sections were examined under a light microscope, and images were captured.

### 2.11. Statistical Analysis

The final results originate from the combination of three biological experiments, each carried out with three technical replicates. Statistical analysis was conducted using Prism 8.0 for Windows (GraphPad software, San Diego, CA, USA). A one-way analysis of variance (ANOVA) was used for the comparison of multiple groups, followed by Tukey’s multiple comparisons test. The statistically significant difference was defined as * *p* < 0.05 and ** *p* < 0.01.

## 3. Results

### 3.1. Isolation and Characterization of the Phages

**Figure 1:**
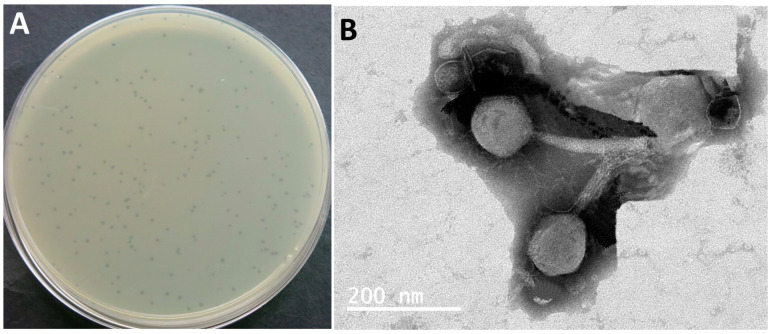
Plaque morphology (**A**) and TEM image of SPEC13 (**B**). The white scale bar represents 200 nm.

The phage that was isolated demonstrated the ability to generate small and distinct plaques on the lawns of *E. coli* O157:H7 cultures and was labeled accordingly (Figure 1A). Upon examination using transmission electron microscopy, it was observed that phage SPEC13 possessed a contractile tail and was classified within the *Ackermannviridae* family (Figure 1B). The measurements of SPEC13’s head were determined to be 83 ± 5 nm, while the contractile tail measured 124 ± 9 nm (Figure 1B).

### 3.2. Spot Test and EOP Assay

Spot tests of the SPEC1–SPEC16 (*n* = 16) phages were conducted on both subgroup O157:H7 (*n* = 18) and non-O157:H7 (*n* = 17) *E. coli* (Table 1). Phage SPEC13 killed 18 out of 18 O157:H7 *E. coli* and 17 out of 17 non-O157:H7 *E. coli* bacteria. Moreover, the comparative analysis of the efficacy of phages targeting *E. coli* O157:H7 as a reference strain revealed the susceptibility of the tested bacterial strains to SPEC13 phage, thus indicating the wide host range specificity of this particular phage (Table 2).

### 3.3. One-Step Growth Curve of SPEC13

**Figure 2:**
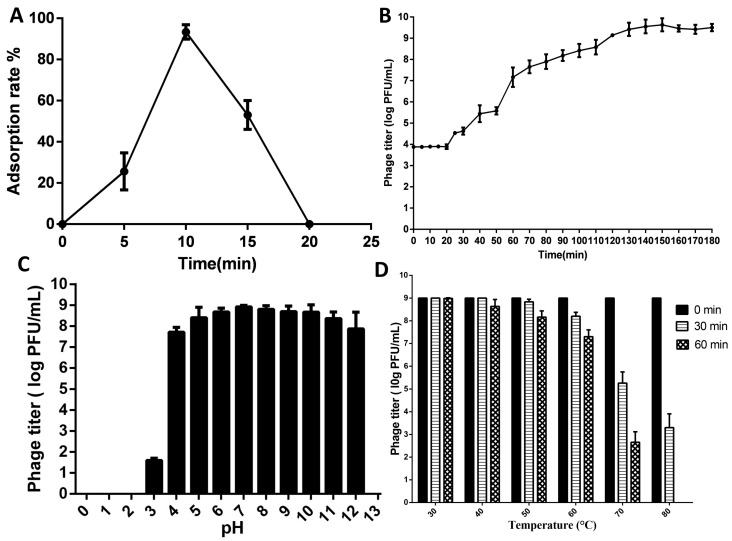
Phage growth curve and stability: absorption rate (**A**), phage titer time effect (**B**), pH Effect (**C**), and temperature and time effect (**D**).

According to Figure 2A, the percentage of SPEC13 phage particles that attached to the host cells rose significantly over a short time period. After 5 min, about a quarter (26%) of the SPEC13 phage had adsorbed. This number then climbed to nearly all (93%) by the 10 min. The examination of the one-step growth curve revealed that the latent phase of phage SPEC13 lasted for 20 min, following which there was a swift escalation in the quantity of phage particles. This phenomenon became evident approximately 150 min later and the number of PFU per infected cell was an average of 139 ± 3 (Figure 2B).

### 3.4. pH and Thermal Stability

The stability of phage SPEC13 when exposed to various pH levels and temperatures was assessed. It was observed that SPEC13 demonstrated a considerable level of stability within the pH range of 4–12, with a stability rate exceeding 85%. Notably, at pH levels between 5 and 10, the stability of SPEC13 surpassed 90% (Figure 2C). Furthermore, SPEC13 exhibited stability across the temperature range of 30 °C to 60 °C, with a stability rate exceeding 80% even at higher temperatures over a period of 60 min, showing minimal loss in infectivity at 60 °C (Figure 2D). Subsequent to a 60-minute incubation at an elevated temperature of 80 °C, no viable SPEC13 particles were detected.

### 3.5. SPEC13 Is a Newly Discovered Phage Belonging to an Unknown Genus

**Figure 3:**
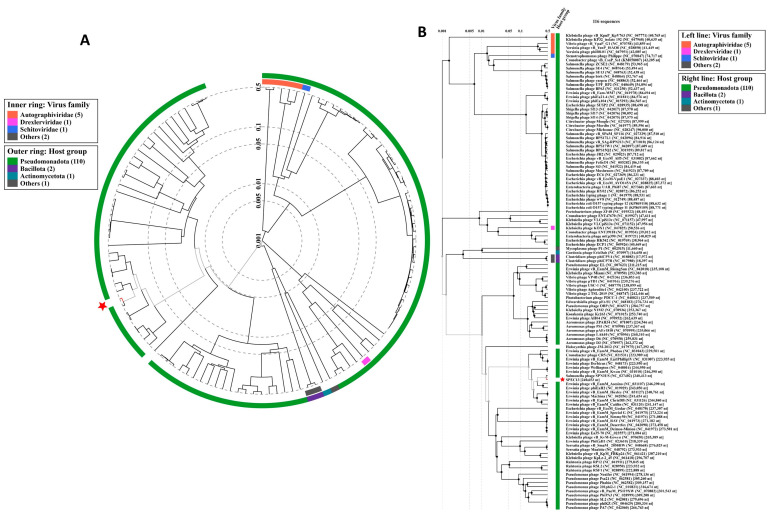
Proteomic phylogeny and whole-genome alignment on ViPTree. (**A**) Circular proteomic tree of SPEC13 and related RefSeq phage genomes on NCBI. (**B**) Rectangular proteomic tree of top matches to phage SPEC13. In both trees, phage SPEC13 is highlighted with a red star.

The comprehensive analysis of the entire genome unveiled that phage SPEC13 harbors a double-stranded DNA genome spanning 240,653 base pairs, exhibiting a GC content of 49.0%. Upon subjecting the genomic sequence to both BLASTn and Viptree analyses, it was ascertained that there exist no substantial resemblances to any previously characterized phages. Consequently, it can be deduced that phage SPEC13 represents a distinctive species situated within the newly categorized ‘*Specvirus*’ genus, belonging to an as yet unclassified family (as illustrated in Figure 3).

**Figure 4:**
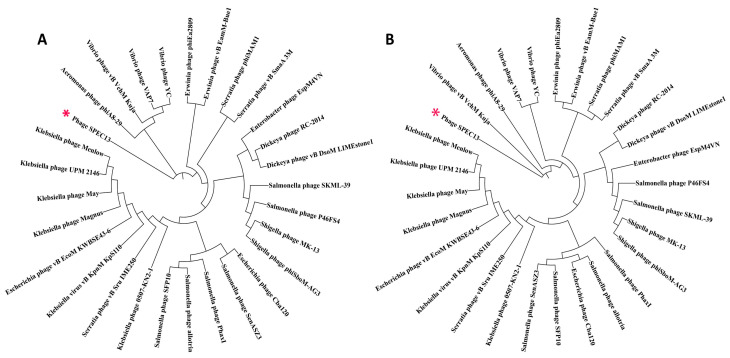
Phylogenetic trees of SPEC13 terminase (large subunit) (**A**) and major capsid protein (**B**). The consensus trees were constructed using the corresponding protein of SPEC13 and related phages with MEGA11. The amino acid sequences were aligned using MUSCLE and a neighbor-joining tree with 1000 bootstraps was constructed. Phages SPEC13 were marked in red star.

Subsequently, a phylogenetic tree utilizing the neighbor-joining method (comprising 1000 bootstrap replicates) was elaborated for both the terminase large subunit protein and the major capsid protein. This analysis aimed to juxtapose the proteins of phage SPEC13 with those of other phages recognized by the MEGA11 software to possess a similar proteome profile (Figure 4). Nevertheless, it was discerned that no semblance existed between SPEC13 and any previously documented phages, neither at the protein level nor at the nucleotide level.

**Figure 5:**
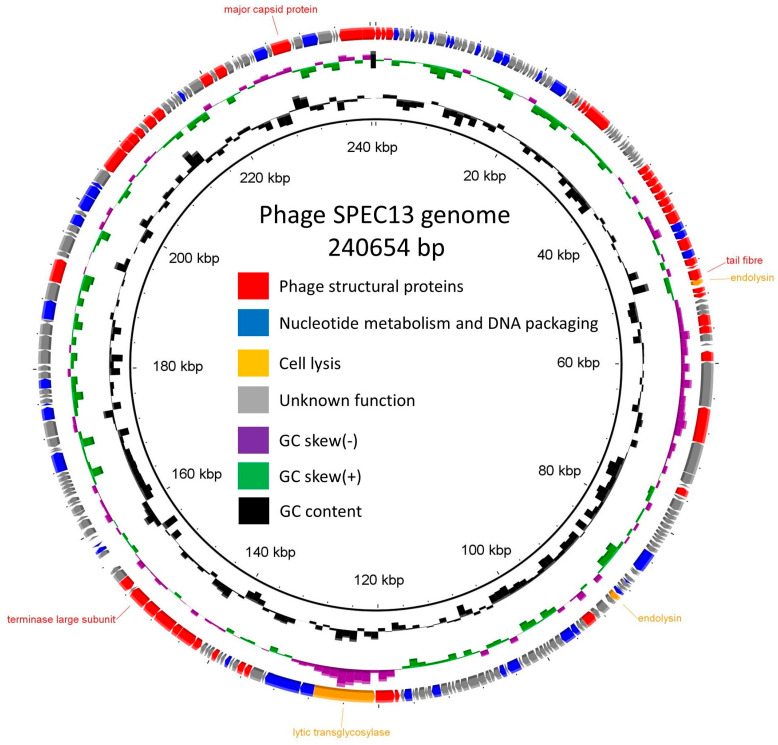
Genome map of SPEC13. Each arrow represents a coding sequence. In red, genes encoding structural proteins; blue, DNA metabolism-associated and packaging proteins; orange, cell-lysis-associated proteins; and in gray, hypothetical proteins constructed by BRIG.jar, 0.95-dist software.

Through meticulous examination of the structural annotation, a total of 262 coding sequences alongside 3 tRNAs were pinpointed (refer to Supplementary Table S1). Notably, out of these coding sequences, 93 were found to encode proteins that could be attributed a putative function, accounting for 35.5% of the total, while the remaining coding sequences (64.5%) were observed to encode hypothetical proteins (Figure 5). Within the subset of genes annotated with functional roles, no indications of a lysogenic lifecycle, antibiotic resistance mechanisms, or virulence-related genes were found. This observation implies that phage SPEC13 potentially holds promise for therapeutic applications, devoid of any genomic elements that could raise concerns from a biomedical standpoint.

### 3.6. Lytic Activity of Phage SPEC13 In Vitro

**Figure 6:**
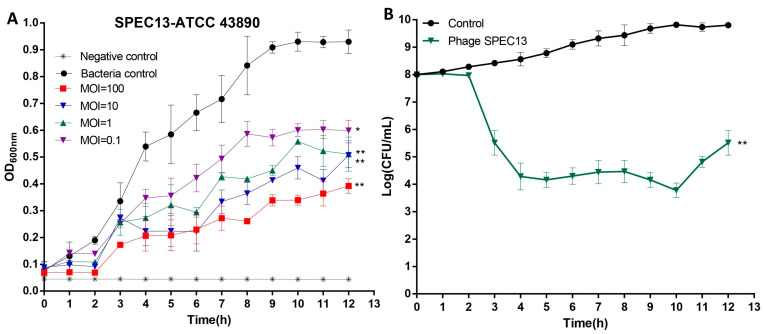
Effect of phage SPEC13 on *E. coli* O157:H7. (**A**) Lytic activity, (**B**) Inhibition of bacterial propagation. Data presented as mean ± SD. *n* = 3. * *p* < 0.05; ** *p* < 0.01.

The investigation presented evidence that, in the event of phage treatment being absent, *E. coli* O157:H7 displayed growth, achieving an optical density of 0.9 at 600 nm following a 3 h period of incubation prior to entering the stationary stage. In contrast, the administration of phage SPEC13 at varying multiplicities of infection (MOIs) of 1, 10, 100 (*p* < 0.01), and 0.1 (*p* < 0.05) markedly inhibited bacterial propagation, as illustrated in Figure 6A.

### 3.7. Inhibition Effect of Phage SPEC13 on E. coli O157:H7 in LB Medium

In the presence of phage SPEC13, a notable decrease in the viable counts of bacterial hosts in the LB medium was observed when compared to the phage-free controls at 37 °C (Figure 6B) starting from 2 h. Following a 4 h phage treatment, there was an approximate 4 log reduction in the viable counts (*p* < 0.01), which exhibited a slight increase after 10 h of incubation.

### 3.8. Effect of Phage on Body Weight and Colon Length

**Figure 7:**
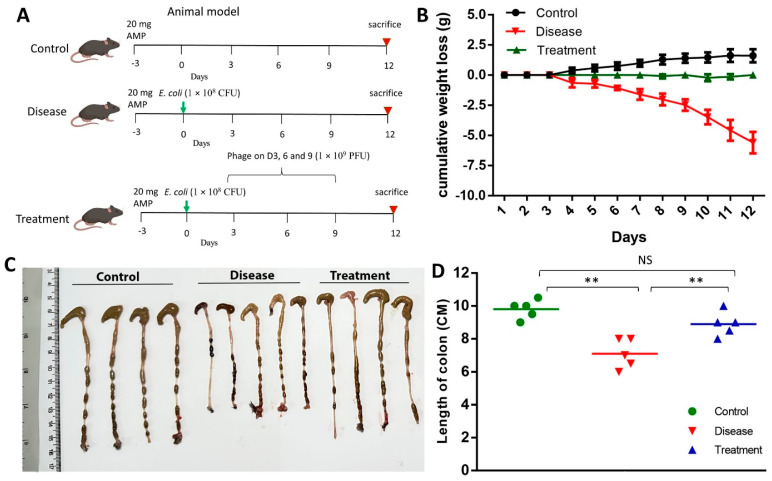
Effect of SPEC13 on mice phenotype. (**A**) Study design, (**B**) Body weight change, (**C**,**D**) Colon length. Data presented as mean ± SD. *n* = 5. ** *p* < 0.01.

It was observed that from the third day onwards, the disease-afflicted mice commenced a decrease in their body weight (Figure 7B). Conversely, the treatment group did not exhibit any reduction in their body weight. Interestingly, while natural weight gain was noticed in the healthy mice, no such trend was observed in the treatment group. The measurement of the colon’s length was conducted as a component of the research investigation (Figure 7C). The colon’s length in the control cohort was documented at approximately 10 cm (Figure 7D). A notably reduced colon length was observed in the group of mice afflicted with the disease, measuring around 7 cm. Conversely, a notably elongated colon, approximately 9 cm in length, was ascertained in the group of mice treated with SPEC13 phage. No statistically significant disparity was detected between the control and treatment groups of mice.

**Figure 8:**
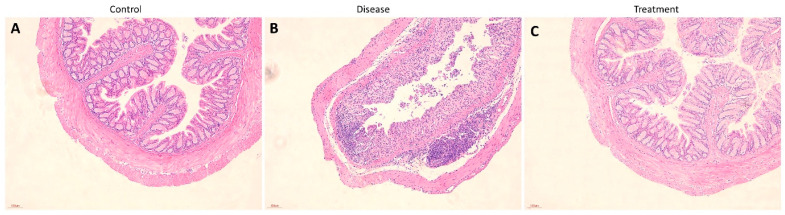
Effect of SPEC13 against disease control. Representative images of rat colonic mucosa of control mice (**A**), disease group (**B**) and bacteriophage treated group (**C**). Scale bar: 100 nm.

A histological analysis conducted in this study delved deeper into the colon tissue samples. A significant improvement caused by SPEC13 was observed in the treatment group mice, in the inflammatory cell infiltration within the colonic tissue, alongside a substantial reduction in colonic edema and mucosal damage ( Figure 8A–C).

### 3.9. Effect of SPEC13 on Fecal Microbe

**Figure 9:**
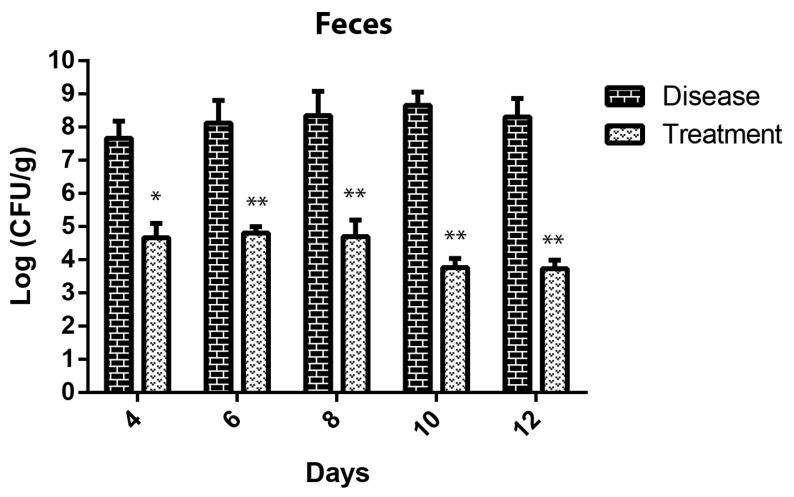
Effect of SPEC13 on reduction in *E.* *coli* O157:H7 in feces. Data presented as mean ± SD. *n* = 5. * *p <* 0.05; ** *p <* 0.01.

Fecal samples were acquired on the 4th, 6th, 8th, and 12th days subsequent to the infection with *E. coli* O157:H7, during which the phage SPEC13 was administered to the mice subjects on the 3rd, 6th, and 9th days. It was observed that the introduction of phage SPEC13 resulted in a substantial reduction in the quantities of *E. coli* O157:H7 present, decreasing to approximately logarithm 5 CFU/g from the 4th day onwards. Particularly noteworthy was the detection of the lowest levels of fecal *E. coli* O157:H7 on the 9th and 12th days, measuring at around logarithm 4 CFU/g, as illustrated in Figure 9.

## 4. Discussion

Of the *E. coli* strains that cause human and animal illness, *E. coli* O157:H7 is the most prevalent type. It can trigger a range of illnesses, from mild diarrhea to potentially fatal hemorrhagic colitis [3]. Phages are particularly good at targeting specific bacteria, leaving helpful ones unharmed. However, they can still lyse most variations within a single harmful bacterial species [33]. Research suggests phages could be a powerful tool in the fight against a wide range of harmful bacteria. In our study of *E. coli* O157:H7, we collected 17 phages. Among them, the results of the spot test and EOP analysis for the SPEC13 phage demonstrate its broad host specificity and high virulence against both O157:H7 and non-O157:H7 *E. coli* strains. The spot test revealed that SPEC13 effectively lysed all 18 O157:H7 and 17 non-O157:H7 *E. coli* strains, indicating its broad-spectrum activity. This finding is significant as it suggests that SPEC13 could be a potential candidate for phage therapy, especially in combating diverse *E. coli* infections, including those caused by antibiotic-resistant strains [34,35]. This broad host specificity is crucial for phage therapy applications, as it ensures that the phage can target a wide array of bacterial strains, thereby enhancing its therapeutic potential [36,37]. Additionally, the high virulence of SPEC13, as indicated by its ability to kill all tested strains, aligns with the need for highly effective phages in phage therapy libraries [38].

The transmission electron microscopy (TEM) analysis revealed that SPEC13 possesses a contractile tail, classifying it within the *Ackermannviridae* family, similar to other phages, like those isolated by Abdulhussein et al., which also exhibited an icosahedral head and contractile tail [39]. The head and tail measurements of SPEC13 (83 ± 5 nm and 124 ± 9 nm, respectively) are within the range reported for other *Ackermannviridae* phages, such as those described by Ngu et al., which had head sizes ranging from 81.2 to 110.77 nm and tail sizes from 115.55 to 132.57 nm [40]. The whole genome analysis of phage SPEC13, revealing a dsDNA genome of 240,653 bp with a GC content of 49.0%, indicates its novelty, as it shows no significant similarities to any previously characterized phages, thus proposing its classification within a new genus, ‘*Specvirus*’, in an unclassified family. This is consistent with findings from other studies where novel phages have been identified and characterized, such as the novel lytic phage pEp_SNUABM_08 infecting *Erwinia pyrifoliae*, which also showed unique genomic characteristics and no close relatives [41]. The neighbor-joining tree constructed for the large terminase and major capsid proteins of SPEC13, showing no similarity to other phages, further underscores its uniqueness, akin to the distinct genomic features observed in phage vB_VpaP_DE10, which infects *Vibrio parahaemolyticus* [42]. The absence of a lysogenic lifecycle, antibiotic resistance, or virulence genes in SPEC13 suggests its potential for safe therapeutic use, paralleling the safety profiles of other phages, like those discussed in the context of phage therapy’s revival due to rising antibiotic resistance [43]. This comprehensive genomic characterization and the potential therapeutic application of SPEC13 highlight the importance of continued phage research and the development of phage therapy infrastructure, as seen in the UK’s efforts to integrate phage therapy into healthcare systems [44].

The observed one-step growth curve of phage SPEC13, with a latent phase lasting 20 min and a subsequent rapid increase in phage particle quantity after approximately 150 min, aligns with findings from various studies on phage infections. The latent period, burst time, and burst size are crucial kinetic parameters that characterize phage–host interactions [45,46]. Moreover, the stability and high burst size of phages, as seen in other studies, suggest that SPEC13 could maintain its efficacy under various environmental conditions, making it a robust tool for both clinical and therapeutic applications [47]. The observed stability of phage SPEC13 across a broad pH range and various temperatures underscores its potential as a robust biocontrol agent. The phage’s stability within the pH range of 4–12 aligns with findings from other studies where phages demonstrated high resistance to extreme pH conditions, particularly alkaline environments [48,49]. The findings of the current research are consistent with those of previous studies; however, the unique characteristics of phages pose a significant challenge when these phages are employed in the treatment of post-gastric organs. The viability and concentration of bacteriophages may be compromised or altered as they traverse the acidic pH of the stomach, which typically ranges from 1.5 to 2.0 [50]. Consequently, further investigation is warranted to address and potentially overcome this constraint. In a recent study, a scalable low-shear membrane emulsification technique was utilized to produce microencapsulated *E. coli* phages. It was observed that the process of microencapsulation provided substantial protection to the phages when exposed for extended periods to a simulated acidic gastric environment [51]. The thermal stability of SPEC13, maintaining over 30 °C to 60 °C, is consistent with the thermal resistance observed in other phages, such as those targeting *Shigella flexneri* and MRSA, which also retained viability and infectivity at elevated temperatures [52,53].

The study on the lytic activity of phage SPEC13 against *E. coli* O157:H7 demonstrated a significant suppression of bacterial growth across various multiplicities of infection (MOIs). This finding aligns with previous research indicating the efficacy of phages in controlling bacterial pathogens. For instance, phage PE-1 demonstrated a similar inhibitory effect on enterotoxigenic *E. coli* (ETEC) K88, significantly reducing bacterial concentration and delaying growth activity [54]. The significant reduction in bacterial growth observed with phage SPEC13 at various MOIs underscores the importance of phage–host dynamics in achieving effective bacterial elimination, as previously noted in studies examining the impact of phage species, bacterial strain, and MOI on bacterial growth dynamics [55]. The observed inhibition effect of phage SPEC13 on *E. coli* O157:H7 in LB medium, particularly the significant reduction in viable bacterial counts at 37 °C, aligns with findings from various studies on phage efficacy against pathogenic *E. coli* strains. The notable decrease in viable counts starting from 1 h and achieving an approximate 4 log reduction after 4 h of phage treatment (*p <* 0.05) demonstrates the potent lytic activity of SPEC13. This result is consistent with the antibacterial effects observed in other phage studies, such as the phage ability to control *S. typhimurium*, *S. enteritidis,* and *E. coli* O157:H7 at different temperatures, including 37 °C [56]. The slight increase in viable counts after 10 h of incubation could be attributed to the emergence of phage-resistant bacterial mutants, a phenomenon documented in phage therapy research [57]. Overall, the detailed characterization of SPEC13 highlights its potential as an effective biocontrol agent against *E. coli* O157:H7, contributing to the growing body of research on phage therapy as an alternative to antibiotics in combating multidrug-resistant bacterial infections.

The study’s findings highlight the promising potential of phage therapy in reversing the effects of *E. coli* infection in mice. The treatment group, administered with SPEC13 phage, demonstrated a significant mitigation of disease symptoms compared to the disease group. Notably, while the disease group exhibited a decline in body weight from the third day onwards, the treatment group maintained stable body weight, indicating the phage’s protective effect against weight loss typically associated with colitis [58]. This aligns with previous research showing that phage therapy can lead to faster recovery and better maintenance of body weight in infected mice compared to treatments group [59]. Additionally, the colon length measurements revealed that the treatment group had a significantly longer colon compared to the disease group, suggesting that SPEC13 phage treatment ameliorated the colonic shortening associated with inflammation [60]. This observation is consistent with other studies where phage therapy has been shown to reduce intestinal injury and delay the onset of colitis [61,62]. These results are in line with the protective effects observed in other phage therapy studies, where phages not only targeted specific bacterial pathogens but also led to reduced inflammation and improved tissue integrity [57,63,64].

The observed effect of phage SPEC13 on fecal *E. coli* O157:H7 in mice demonstrates the potential efficacy of phage therapy in reducing pathogenic bacterial loads. The administration of SPEC13 on the 3rd, 6th, and 9th days post-infection led to a significant reduction in *E. coli* O157:H7 levels, with a notable decrease to approximately logarithm 5 CFU/g from the 4th day onwards. This reduction aligns with findings from other studies that highlight the effectiveness of phage therapy in managing enteric infections. For instance, a study comparing phage therapy to antibiotic treatment in a mouse model of STEC O157:H7 infection found that phage treatment not only provided high survival rates but also facilitated faster recovery and better maintenance of gut microbiota homeostasis compared to antibiotics [65]. Additionally, the use of phages has been shown to significantly decrease bacterial loads and improve health outcomes in various models, such as the reduction in ETEC in piglet intestinal cells and the effective eradication of biofilm-embedded *E. coli* cells in a rat model [66]. The recorded minimal levels of fecal *E. coli* O157:H7 on the 9th and 12th days, quantified at approximately log 4 CFU/g, highlight the enduring effectiveness of SPEC13 over time, in line with the prolonged advantages documented in other phage therapy investigations [67,68]. It is important to emphasize that the application of the novel phage SPEC13 did not entirely eradicate the *E. coli* O157:H7. This could lead to the development of phage resistance by *E. coli* O157:H7 and potentially result in adverse health consequences [69]. Conversely, the dosages of SPEC13 may influence the survivability of fecal *E. coli* O157:H7. For instance, a study demonstrated that a high dose of lytic phage (10^9^ PFU/mL) administered intraperitoneally to Swiss mice infected with a lethal dose of *E. coli* (10^9^ CFU/mL) resulted in 100% survival, effectively suppressing *E. coli* lethality [70]. Consequently, forthcoming research endeavors are essential to ascertain the extent of negative health implications and explore potential strategies to combat such phage resistance if it arises. Overall, these findings reinforce the promise of phage therapy as a viable alternative to antibiotics, particularly in the context of rising antibiotic resistance and the need for targeted, microbiota-sparing treatments.

While the study on phage SPEC13 against *E. coli* O157:H7 showcases promising results, several limitations must be considered. The acidic pH of the stomach, ranging from 1.5 to 2.0, may compromise phage viability, suggesting a need for strategies like microencapsulation to enhance stability. Additionally, the emergence of phage-resistant bacterial mutants, observed as a slight increase in viable counts after 10 h, poses a significant challenge test in vitro. Incomplete eradication of *E. coli* O157:H7 in animal models raises concerns about potential resistance development, highlighting the need for further research on optimal dosing and combination therapies. It is also crucial to study the effects of SPEC13 across different genders and age groups of animals to ensure its broad efficacy and safety. Despite its broad-spectrum activity, careful consideration of impacts on beneficial microbiota is essential. Addressing these limitations through targeted research is vital for the successful therapeutic use of SPEC13.

## 5. Conclusions

In this study, the novel *E. coli* O157:H7 phage SPEC13 was identified, isolated, and characterized, and its antimicrobial potential was evaluated. SPEC13 significantly inhibited *E. coli* O157:H7 growth across various MOIs, aligning with findings from other effective phages, supporting its use as an antibacterial agent. Our experimental results suggest that SPEC13 lysed all tested O157:H7 and non- O157:H7 *E. coli* strains, indicating broad host specificity and high virulence, crucial for phage therapy applications. In vivo studies revealed significant mitigation of colitis symptoms and reduction in fecal *E. coli* O157 loads, demonstrating SPEC13’s therapeutic potential.

## Figures and Tables

**Table 1:** Spot test results of 16 phages.

Bacteria (Number)	Serogroup	Phage															
		SPEC1	SPEC 2	SPEC 3	SPEC 4	SPEC 5	SPEC 6	SPEC7	LPEC8	SPEC9	SPEC10	SPEC11	SPEC12	SPEC13	SPEC14	SPEC15	SPEC16
*E. coli* (*n* = 18)	O157:H7	11	9	8	13	11	10	11	10	9	6	9	5	18	7	11	13
*E. coli* (*n* = 17)	non-O157:H7	9	10	6	8	9	4	6	8	11	3	5	2	17	2	6	7

**Table 2:** Host range of phage SPEC13.

Bacteria	Strains	Serotype	Drug Resistance	SPEC13/ Spot Test	SPEC13/ EOP	Sources
*E. coli*	ATCC 43890	O157:H7	/	+	1	ATCC (Host)
*E. coli*	KCJ4201	O157:H7	/	+	1	[3]
*E. coli*	EDL933	O157:H7	/	+	1	[3]
*E. coli*	DH5α	/	/	+	1	L.S.
*E. coli*	BL21	/	/	+	1	L.S.
*E. coli*	Trans 10	/	/	+	1	L.S.
*E. coli*	FEC6	O157:H7	/	+	1	L.S.
*E. coli*	FEC9	O157:H7	CIP/AMP	+	1	L.S.
*E. coli*	FEC10	O157:H7	CIP/AMP	+	1	L.S.
*E. coli*	FEC16	/	/	+	1	L.S.
*E. coli*	FEC17	/	/	+	1	L.S.
*E. coli*	FEC29	/	/	+	1	L.S.
*E. coli*	FEC28	/	/	+	0.9	L.S.
*E. coli*	FEC14	O157:H7	CIP/AMP/KAN/PLC	+	0.9	L.S.
*E. coli*	FEC24	/	/	+	0.85	L.S.
*E. coli*	FEC3	O157:H7	/	+	0.8	L.S.
*E. coli*	FEC15	O157:H7	CIP/AMP/KAN/STR	+	0.8	L.S.
*E. coli*	FEC12	O157:H7	CIP/AMP/GEN/KAN	+	0.8	L.S.
*E. coli*	FEC22	/	/	+	0.8	L.S.
*E. coli*	FEC1	O157:H7	/	+	0.7	L.S.
*E. coli*	FEC2	O157:H7	/	+	0.7	L.S.
*E. coli*	FEC13	O157:H7	CIP/AMP/KAN/CHL	+	0.7	L.S.
*E. coli*	FEC4	O157:H7	/	+	0.6	L.S.
*E. coli*	FEC26	/	/	+	0.6	L.S.
*E. coli*	FEC5	O157:H7	/	+	0.5	L.S.
*E. coli*	FEC7	O157:H7	/	+	0.4	L.S.
*E. coli*	FEC11	O157:H7	CIP/AMP/GEN/KAN/STR/CHL/PCS	+	0.4	L.S.
*E. coli*	FEC18	/	/	+	0.4	L.S.
*E. coli*	FEC8	O157:H7	/	+	0.3	L.S.
*E. coli*	FEC19	/	/	+	0.3	L.S.
*E. coli*	FEC20	/	/	+	0.3	L.S.
*E. coli*	FEC21	/	/	+	0.2	L.S.
*E. coli*	FEC23	/	/	+	0.2	L.S.
*E. coli*	FEC25	/	/	+	0.1	L.S.
*E. coli*	FEC27	/	/	+	0.1	L.S.
*S. Enteritidis*	ATCC 13076	/	/	-	0	ATCC
*S. Enteritidis*	SGSC 4901	/	/	-	0	L.S.
*S. Typhimurium*	ATCC 14028	/	/	-	0	ATCC
*S. Typhimurium*	ATCC 13311	/	/	-	0	ATCC
*Staphylococcus aureus*	ATCC 29213	/	/	-	0	ATCC
*Listeria monocytogenes*	ATCC 19115	/	/	-	0	ATCC
*Bacteroides fragilis*	ATCC 43858	/	/	-	0	ATCC
*Pseudomonas aeruginosa*	ATCC 7853	/	/	-	0	ATCC
*Fusobacterium*	ATCC 10953	/	/	-	0	ATCC

Note: EOP 0.5 to 1.0, high efficiency; EOP 0.2 to <0.5, moderate efficiency; 0.001 to < 0.2, low efficiency; and <0.001, inefficient. ATCC, American Type Culture Collection; AMP, Ampicillin; CIP, Ciprofloxacin; CHL, Chloramphenicol; GEN, Gentamicin; KAN, Kanamycin; PCS, Pediatric Compound Sulfamethoxazole; STR, Streptomycin; L.S., Lab stock.

## Data Availability

The original contributions presented in the study are included in the article/Supplementary Materials, further inquiries can be directed to the corresponding authors.

## Supplementary Materials

The following supporting information can be downloaded at: https://www.mdpi.com/article/10.3390/biomedicines12092036/s1, Table S1: Functional annotation of SPEC13 CDSs using BLASTP and conserved domains.

## References

[1] Othman S.M., Sheet O.H., Alsanjary R.A. (2022). Isolation and identification of *Escherichia coli* O157:H7 isolated from Veal Meats and Butchers’ Shops in Mosul city, Iraq. J. Appl. Vet. Sci..

[2] Kolodziejek A.M., Minnich S.A., Hovde C.J. (2022). *Escherichia coli* 0157:H7 virulence factors and the ruminant reservoir. Curr. Opin. Infect. Dis..

[3] Zhang Y., Zou G., Islam M.S., Liu K., Xue S., Song Z., Ye Y., Zhou Y., Shi Y., Wei S. (2023). Combine thermal processing with polyvalent phage LPEK22 to prevent the *Escherichia coli* and *Salmonella enterica* contamination in food. Food Res. Int..

[4] Lim J.Y., Yoon J., Hovde C.J. (2010). A brief overview of *Escherichia coli* O157:H7 and its plasmid O157. J. Microbiol. Biotechnol..

[5] Hossain M.M.K., Islam M.S., Uddin M.S., Rahman A.T.M.M., Ud-Daula A., Islam M.A., Rubaya R., Bhuiya A.A., Alim M.A., Jahan N. (2023). Isolation, Identification and Genetic Characterization of Antibiotic Resistant *Escherichia coli* from Frozen Chicken Meat Obtained from Supermarkets at Dhaka City in Bangladesh. Antibiotics.

[6] Munns K.D., Selinger L.B., Stanford K., Guan L., Callaway T.R., McAllister T.A. (2015). Perspectives on super-shedding of *Escherichia coli* O157:H7 by cattle. Foodborne Pathog. Dis..

[7] Ma J., Ibekwe A.M., Yi X., Wang H., Yamazaki A., Crowley D.E., Yang C.H. (2011). Persistence of *Escherichia Coli* O157:H7 and Its Mutants in Soils. PLoS ONE.

[8] Alam M.J., Zurek L. (2004). Association Of *Escherichia coli* O157:H7 with Houseflies on a Cattle Farm. Appl. Environ. Microbiol..

[9] Klous G., Huss A., Heederik D.J.J., Coutinho R.A. (2016). Human–livestock contacts and their relationship to transmission of zoonotic pathogens, a systematic review of literature. One Health.

[10] Ariyanti T., Rachmawati F., Noor S.M., Suhaemi (2022). Contamination of *Escherichia coli* O157:H7 in Milk and Its Dairy Products in Depok, Cianjur, Sukabumi and Bandung. IOP Conf. Ser. Earth Environ. Sci..

[11] Sancak Y.C., Sancak H., İşleyici Ö., Durmaz H. (2015). Presence of *Escherichia coli* O157 and O157:H7 in raw milk and Van herby cheese. Bull. Vet. Inst. Pulawy.

[12] Bull M.B., Cohen C.A., Leung N.H.L., Valkenburg S.A. (2021). Universally Immune: How Infection Permissive Next Generation Influenza Vaccines May Affect Population Immunity and Viral Spread. Viruses.

[13] Brás A., Braz M., Martinho I., Duarte J., Pereira C., Almeida A. (2024). Effect of Bacteriophages against Biofilms of *Escherichia coli* on Food Processing Surfaces. Microorganisms.

[14] Ariyanti T., Noor S.M., Suhaemi, Rachmawati F. (2022). Antimicrobial Resistance Pattern of *Escherichia coli* O157:H7 Isolated from Cattle in West Java, Indonesia. IOP Conf. Ser. Earth Environ. Sci..

[15] Nart P., Naylor S.W., Huntley J.F., McKendrick I.J., Gally D.L., Low J.C. (2008). Responses of Cattle to Gastrointestinal Colonization by *Escherichia Coli* O157:H7. Infect. Immun..

[16] Arthur T.M., Bosilevac J.M., Brichta-Harhay D.M., Guerini M.N., Kalchayanand N., Shackelford S.D., Wheeler T.L., Koohmaraie M. (2007). Transportation and Lairage Environment Effects on Prevalence, Numbers, and Diversity of *Escherichia coli* O157:H7 on Hides and Carcasses of Beef Cattle at Processing. J. Food Prot..

[17] Grami E., Badawy S., Kiljunen S., Saidi N., Skurnik M. (2023). Characterization and genome analysis of *Escherichia* phage fBC-Eco01, isolated from wastewater in Tunisia. Arch. Virol..

[18] Yamaki S., Yamazaki K., Kawai Y. (2022). Broad host range bacteriophage, EscoHU1, infecting *Escherichia coli* O157:H7 and *Salmonella enterica*: Characterization, comparative genomics, and applications in food safety. Int. J. Food Microbiol..

[19] Bumunang E.W., Zaheer R., Niu D., Narvaez-Bravo C., Alexander T., McAllister T.A., Stanford K. (2023). Bacteriophages for the Targeted Control of Foodborne Pathogens. Foods.

[20] Mizoguchi K., Morita M., Fischer C.R., Yoichi M., Tanji Y., Unno H. (2003). Coevolution of Bacteriophage PP01 And *Escherichia Coli* O157:H7 in Continuous Culture. Appl. Environ. Microbiol..

[21] Henrique I.d.M., Sacerdoti F., Ferreira R.L., Henrique C., Amaral M.M., Piazza R.M.F., Luz D. (2022). Therapeutic Antibodies Against Shiga Toxins: Trends and Perspectives. Front. Cell. Infect. Microbiol..

[22] Zhang Y., Liao Y.-T., Salvador A., Sun X., Wu V.C.H. (2020). Prediction, Diversity, and Genomic Analysis of Temperate Phages Induced from Shiga Toxin-Producing *Escherichia coli* Strains. Front. Microbiol..

[23] Islam M.S., Zhou Y., Liang L., Nime I., Liu K., Yan T., Wang X., Li J. (2019). Application of a Phage Cocktail for Control of *Salmonella* in Foods and Reducing Biofilms. Viruses.

[24] Guo Y., Li J., Islam M.S., Yan T., Zhou Y., Liang L., Connerton I.F., Deng K., Li J. (2021). Application of a novel phage vB_SalS-LPSTLL for the biological control of *Salmonella* in foods. Food Res. Int..

[25] Islam M.S., Hu Y., Mizan M.F.R., Yan T., Nime I., Zhou Y., Li J. (2020). Characterization of *Salmonella* Phage LPST153 That Effectively Targets Most Prevalent *Salmonella* Serovars. Microorganisms.

[26] Islam M.S., Zhou Y., Liang L., Nime I., Yan T., Willias S.P., Mia M.Z., Bei W., Connerton I.F., Fischetti V.A. (2020). Application of a Broad Range Lytic Phage LPST94 for Biological Control of *Salmonella* in Foods. Microorganisms.

[27] Islam M.S., Nime I., Pan F., Wang X. (2023). Isolation and characterization of phage ISTP3 for bio-control application against drug-resistant *Salmonella*. Front. Microbiol..

[28] Fister S., Fuchs S., Stessl B., Schoder D., Wagner M., Rossmanith P. (2016). Screening and characterisation of bacteriophage P100 insensitive Listeria monocytogenes isolates in Austrian dairy plants. Food Control.

[29] Islam M.S., Yang X., Euler C.W., Han X., Liu J., Hossen M.I., Zhou Y., Li J. (2021). Application of a novel phage ZPAH7 for controlling multidrug-resistant *Aeromonas hydrophila* on lettuce and reducing biofilms. Food Control.

[30] Islam M.S., Raz A., Liu Y., Elbassiony K.R.A., Dong X., Zhou P., Zhou Y., Li J. (2019). Complete Genome Sequence of *Aeromonas* Phage ZPAH7 with Halo Zones, Isolated in China. Microbiol. Resour. Announc..

[31] Kotwal S., Kaul S., Sharma P., Gupta M., Shankar R., Jain M., Dhar M.K. (2016). De Novo Transcriptome Analysis of Medicinally Important Plantago ovata Using RNA-Seq. PLoS ONE.

[32] Zhang M., Qiu X., Zhang H., Yang X., Hong N., Yang Y., Chen H., Yu C. (2014). *Faecalibacterium prausnitzii* Inhibits Interleukin-17 to Ameliorate Colorectal Colitis in Rats. PLoS ONE.

[33] Almeida A., Cunha A., Gomes N.C.M., Alves E., Costa L., Faustino M.A.F. (2009). Phage therapy and photodynamic therapy: Low environmental impact approaches to inactivate microorganisms in fish farming plants. Mar. Drugs.

[34] Hallajzadeh M., Mojtahedi A., Amirmozafari N., Pirhajati Mahabadi V. (2020). Characterizing a Lytic Bacteriophage Infecting Methicillin-Resistant *Staphylococcus aureus* (MRSA) Isolated from Burn Patients. Arch. Clin. Infect. Dis..

[35] Tian Y., Liang T., Zhu P., Chen Y., Chen W., Du L., Wu C., Wang P. (2019). Label-Free Detection of *E. coli* O157:H7 DNA Using Light-Addressable Potentiometric Sensors with Highly Oriented ZnO Nanorod Arrays. Sensors.

[36] Wu C., Li D., Jiang Q., Gan N. (2023). A Paper-Chip-Based Phage Biosensor Combined with a Smartphone Platform for the Quick and On-Site Analysis of *E. coli* O157:H7 in Foods. Chemosensors.

[37] Ateba C.N., Mbewe M. (2011). Detection of *Escherichia coli* O157:H7 virulence genes in isolates from beef, pork, water, human and animal species in the northwest province, South Africa: Public health implications. Res. Microbiol..

[38] Zhang D., Coronel-Aguilera C.P., Romero P.L., Perry L., Minocha U., Rosenfield C., Gehring A.G., Paoli G.C., Bhunia A.K., Applegate B. (2016). The Use of a Novel NanoLuc -Based Reporter Phage for the Detection of *Escherichia coli* O157:H7. Sci. Rep..

[39] Abdulhussein A.A., Abdulsattar B.O. (2023). Identification and Characterization of a Bacteriophage with Lytic Activity against Multidrug Resistant *E. coli*. Al-Mustansiriyah J. Sci..

[40] Ngu N., Loc H., Nhan N., Huan P., Anh L., Xuan N. (2020). Isolation and characterization of bacteriophages against *Escherichia coli* isolates from chicken farms. Adv. Anim. Vet. Sci..

[41] Li F., Li L., Na S., Zhao J., Liu F., Liu P., Li Y., Li M., Lei M., Zhang D. (2023). Isolation, characterization and genomic analysis of a novel phage IME178 with lytic activity against *Escherichia coli*. Microb. Pathog..

[42] Ye Y., Chen H., Huang Q., Huang S., He J., Zhang J., Wu Q., Li X., Hu W., Yang M. (2022). Characterization and Genomic Analysis of Novel *Vibrio parahaemolyticus* Phage vB_VpaP_DE10. Viruses.

[43] Zhu Y., Shang J., Peng C., Sun Y. (2022). Phage family classification under Caudoviricetes: A review of current tools using the latest ICTV classification framework. Front. Microbiol..

[44] Konieczka T.A., Wolyniak M.J. (2022). Isolation and characterization of the novel Mycobacterium smegmatis phage Dawnguard. FASEB J..

[45] Zaburlin D., Quiberoni A., Mercanti D. (2017). Changes in environmental conditions modify infection kinetics of dairy phages. Food Environ. Virol..

[46] Blazanin M., Vasen E., Jolis C.V., An W., Turner P.E. (2023). Theoretical validation of growth curves for quantifying phage-bacteria interactions. bioRxiv.

[47] Park D.-W., Lim G.-y., Lee Y.-d., Park J.-H. (2020). Characteristics of lytic phage vB_EcoM-ECP26 and reduction of shiga-toxin producing *Escherichia coli* on produce romaine. Appl. Biol. Chem..

[48] Tomat D., Gonzalez A., Aquili V., Casabonne C., Quiberoni A. (2022). Physicochemical characterization of ten newly isolated phages against the foodborne pathogen *Shigella flexneri*. J. Food Process. Preserv..

[49] Abd-Allah I.M., El-Housseiny G.S., Alshahrani M.Y., El-Masry S.S., Aboshanab K.M., Hassouna N.A. (2022). An anti-MRSA phage from raw fish rinse: Stability evaluation and production optimization. Front. Cell. Infect. Microbiol..

[50] Fujimori S. (2020). Gastric acid level of humans must decrease in the future. World J. Gastroenterol..

[51] Vinner G.K., Richards K., Leppanen M., Sagona A.P., Malik D.J. (2019). Microencapsulation of Enteric Bacteriophages in a pH-Responsive Solid Oral Dosage Formulation Using a Scalable Membrane Emulsification Process. Pharmaceutics.

[52] Balestreri C., Schroeder D.C., Sampedro F., Marqués G., Palowski A., Urriola P.E., van de Ligt J.L., Yancy H.F., Shurson G.C. (2024). Unexpected thermal stability of two enveloped megaviruses, Emiliania huxleyi virus and African swine fever virus, as measured by viability PCR. Virol. J..

[53] Kering K.K., Zhang X., Nyaruaba R., Yu J., Wei H. (2020). Application of adaptive evolution to improve the stability of bacteriophages during storage. Viruses.

[54] Hanif S., Das R., Chavan B., Shah S., Bajpai U., Ahmed S. (2023). P10 Phage-encoded lysins as promising antibacterials against uropathogenic *Escherichia coli*. JAC-Antimicrob. Resist..

[55] Alexyuk P., Bogoyavlenskiy A., Alexyuk M., Akanova K., Moldakhanov Y., Berezin V. (2022). Isolation and characterization of lytic bacteriophages active against clinical strains of *E. coli* and development of a phage antimicrobial cocktail. Viruses.

[56] Duc H.M., Zhang Y., Hoang S.M., Masuda Y., Honjoh K.-I., Miyamoto T. (2023). The use of phage cocktail and various antibacterial agents in combination to prevent the emergence of phage resistance. Antibiotics.

[57] Zhao P., Meng X. (2023). Characterization of antibacterial activity of lytic bacteriophage PE-1 for biological control of *Escherichia coli* K88 in vitro. Food Sci. Anim. Resour..

[58] Jackson K., Galipeau H., Hann A., Coombes B., Hosseinidoust Z., Verdu E. (2023). A32 phage treatment delays onset of crohn’s-associated *E. coli* driven colitis in mice colonized with a defined microbiota. J. Can. Assoc. Gastroenterol..

[59] Wang Y., Subedi D., Li J., Wu J., Ren J., Xue F., Dai J., Barr J.J., Tang F. (2022). Phage Cocktail Targeting STEC O157:H7 Has Comparable Efficacy and Superior Recovery Compared with Enrofloxacin in an Enteric Murine Model. Microbiol. Spectr..

[60] Marongiu L., Burkard M., Lauer Ulrich M., Hoelzle Ludwig E., Venturelli S. (2022). Reassessment of Historical Clinical Trials Supports the Effectiveness of Phage Therapy. Clin. Microbiol. Rev..

[61] Pessina B., Guarnieri V., Scarallo L. (2023). Entering the era of phage therapy: A ‘happy hour’ for inflammatory bowel diseases. Allergy.

[62] Nishio J., Negishi H., Yasui-Kato M., Miki S., Miyanaga K., Aoki K., Mizusawa T., Ueno M., Ainai A., Muratani M. (2021). Identification and characterization of a novel *Enterococcus* bacteriophage with potential to ameliorate murine colitis. Sci. Rep..

[63] Islam M.S., Fan J., Pan F. (2023). The power of phages: Revolutionizing cancer treatment. Front. Oncol..

[64] Suda T., Hanawa T., Tanaka M., Tanji Y., Miyanaga K., Hasegawa-Ishii S., Shirato K., Kizaki T., Matsuda T. (2022). Modification of the immune response by bacteriophages alters methicillin-resistant *Staphylococcus aureus* infection. Sci. Rep..

[65] Wortelboer K., de Jonge P.A., Scheithauer T.P.M., Attaye I., Marleen Kemper E., Nieuwdorp M., Herrema H. (2023). Phage-microbe dynamics after sterile faecal filtrate transplantation in individuals with metabolic syndrome: A double-blind, randomised, placebo-controlled clinical trial assessing efficacy and safety. Nat. Commun..

[66] Laforêt F., Antoine C., Lebrun S., Gonza I., Goya-Jorge E., Douny C., Duprez J.-N., Scippo M.-L., Taminiau B., Daube G. (2023). Impact Assessment of vB_KpnP_K1-ULIP33 Bacteriophage on the Human Gut Microbiota Using a Dynamic In Vitro Model. Viruses.

[67] Abdelaziz A.A., Abo Kamer A.M., Nosair A.M., Al-Madboly L.A. (2023). Exploring the potential efficacy of phage therapy for biocontrol of foodborne pathogenic extensively drug-resistant *Escherichia coli* in gastrointestinal tract of rat model. Life Sci..

[68] Azam A.H., Sato K., Miyanaga K., Nakamura T., Ojima S., Kondo K., Tamura A., Yamashita W., Tanji Y., Kiga K. (2024). Selective bacteriophages reduce the emergence of resistant bacteria in bacteriophage-antibiotic combination therapy. Microbiol. Spectr..

[69] McGee L.W., Barhoush Y., Shima R., Hennessy M. (2023). Phage-resistant mutations impact bacteria susceptibility to future phage infections and antibiotic response. Ecol. Evol..

[70] Leta A., Yohannes M., Kassa T. (2017). Assessment of therapeutic potential of bacteriophages to control *Escherichia coli* infection in Swiss mice model. Ethiop. J. Appl. Sci. Technol..

