# Supplementary material for: Isolation and Characterization of Novel Escherichia coli O157:H7 Phage SPEC13 as a Therapeutic Agent for E. coli Infections In Vitro and In Vivo
Source: Biomedicines. 2024 Sep 6;12(9):2036. doi: 10.3390/biomedicines12092036 (PMC11428821; doi:10.3390/biomedicines12092036)
Supplement: Supplementary file 1 [file biomedicines-12-02036-s001.zip › biomedicines-3126525-supplementary.pdf]

biomedicines

MDPI

Supplementary Materials

Isolation and Characterization of Novel *Escherichia coli* O157:H7 Phage SPEC13 as a Therapeutic Agent for *E. coli* Infections In Vitro and In Vivo

Md. Sharifull Islam [1,†], Jie Fan [2,†], Md Suzauddula [3], Ishatur Nime [4] and Fan Pan [1,*]

[1] Center for Cancer Immunology, Institute of Biomedicine and Biotechnology, Shenzhen Institute of Advanced Technology, Chinese Academy of Sciences, Shenzhen 518055, China; smbgb101287@yahoo.com

[2] Department of Pathology, School of Basic Medicine, Henan University of Science and Technology, 263 Kaiyuan Avenue, Luoyang 471023, China; fanjie277185479@gmail.com

[3] College of Agriculture and Natural Resources, National Chung Hsing University, Taichung 40227, Taiwan; mdsuzauddula@gmail.com

[4] Key Laboratory of Environment Correlative Dietology, College of Food Science and Technology, Huazhong Agricultural University, Wuhan 430070, China; smbgb101287@gmail.com

* Correspondence: fan.pan@siat.ac.cn

† The authors contributed equally to this work.

Table S1. Functional annotation of SPEC13 CDSs using BLASTP and conserved domains

ORF	Gene Positions	Functions	Name	Accession
**1**	1-663	putative virion structural protein [Erwinia phage vB_EamM_Stratton]		
**2**	671-1193	virion structural protein [Erwinia phage vB_EamM_Asesino]		
**3**	1191-2078	putative virion structural protein [Erwinia phage vB_EamM_Stratton]		
**4**	2078-2728	RuvC-like Holliday junction resolvase [Salmonella phage SPLA5c]	RuvC-like superfamily	cl21482
**5**	2731-3303	hypothetical protein [Escherichia phage vB_EcoM_Lh1B]		
**6**	3401-4039	hypothetical protein [Escherichia phage vB_EcoM_EC001]		
**7**	4039-4521	acetyltransferase [Erwinia phage vB_EamM_Huxley]	Acetyltransf_1	pfam00583
**8**	4539-5015	hypothetical protein [Escherichia phage vB_EcoM_Lh1B]		
**9**	5018-5407	hypothetical protein [Salmonella phage JN03]		
**10**	5498-6082	hypothetical protein [Salmonella phage MET_P1_082_240]		
**11**	6199-6798	DprA-like DNA recombination-mediator protein [Salmonella phage SPLA1a]		
**12**	6829-7101	hypothetical protein [Escherichia phage vB_EcoM_Lh1B]		
**13**	7112-8245	hypothetical protein SPFM4_00083 [Salmonella phage SPFM4]		
**14**	8245-8706	GNAT family N-acetyltransferase [Erwinia phage pEa_SNUABM_8]	Acetyltransf_1	pfam00583
**15**	8706-9170	N-acetyltransferase [Salmonella phage SPAsTU]	Acetyltransf_1	pfam00583
**16**	9172-9780	hypothetical protein [Erwinia phage pEa_SNUABM_29]		
**17**	9726-10097	hypothetical protein [Salmonella phage SPN3US]		
**18**	10111-10707	hypothetical protein [Salmonella phage SaP7]		
**19**	10865-11623	hypothetical protein [Enterobacteria phage SEGD1]		
**20**	11738-12361	putative thymidylate kinase [Salmonella phage JN03]	NK superfamily	cl17190
**21**	12526-12891	putative transcriptional regulator [Salmonella phage SaP7]	HTH_XRE superfamily	cl22854
**22**	12903-13292	hypothetical protein [Salmonella phage JN03]		
**23**	13315-13971	hypothetical protein [Enterobacteria phage SEGD1]		

ORF	Gene Positions	Functions	Name	Accession
**24**	14061-15059	N-acyltransferase superfamily protein [Escherichia phage vB_EcoM_EC001]	Acetyltransf_1	pfam00583
**25**	15066-15884	DNA methyltransferase [Salmonella phage SPN3US]	dam	TIGR00571
**26**	15894-16697	hypothetical protein [Salmonella phage SPLA1a]		
**27**	16707-17519	hypothetical protein [Salmonella phage JN03]		
**28**	17509-17787	hypothetical protein [Salmonella phage STsAS]		
**29**	17828-18310	hypothetical protein [Salmonella phage MET_P1_082_240]		
**30**	18407-18742	hypothetical protein [Erwinia phage pEa_SNUABM_10]		
**31**	18720-19046	hypothetical protein pEaSNUABM6_00127 [Erwinia phage pEa_SNUABM_6]		
**32**	19128-19472	hypothetical protein [Erwinia phage pEa_SNUABM_43]		
**33**	19530-20054	putative HslV-like ATP-dependent protease [Erwinia phage pEa_SNUABM_43]		
**34**	20051-20608	hypothetical protein [Erwinia phage Machina]		
**35**	20652-21350	hypothetical protein [Salmonella phage SPLA1a]		
**36**	21350-21559	hypothetical protein [Escherichia phage vB_EcoM_EC001]		
**37**	21559-23319	ATP-dependent protease [Salmonella phage MET_P1_082_240]	hslU superfamily	cl35293
**38**	23448-23780	hypothetical protein [Salmonella phage STsAS]		
**39**	23789-24796	hypothetical protein [Salmonella phage SPLA1a]		
**40**	24741-25124	DUF488 domain containing protein [Erwinia phage pEa_SNUABM_43]	DUF488 superfamily	cl01246
**41**	25136-25531	hypothetical protein [Salmonella phage SaP7]		
**42**	25602-26135	virion structural protein [Salmonella phage SPLA1a]		
**43**	26135-26605	minor tail protein [Salmonella phage PMBT28]		
**44**	26609-29626	tail protein [Salmonella phage SPLA5c]		
**45**	29754-30086	hypothetical protein [Enterobacteria phage SEGD1]		
**46**	30130-30414	hypothetical protein [Salmonella phage SaP7]		
**47**	30442-30960	hypothetical protein [Salmonella phage SPN3US]		

ORF	Gene Positions	Functions	Name	Accession
**48**	30971-31291	hypothetical protein [Escherichia phage vB_EcoM_EC001]		
**49**	31291-31674	hypothetical protein [Proteus phage 7]		
**50**	32050-32355	hypothetical protein [Salmonella phage SaP7]		
**51**	32421-32822	hypothetical protein [Salmonella phage JN03]		
**52**	32900-33703	hypothetical protein [Escherichia phage vB_EcoM_EC001]		
**53**	33693-33920	hypothetical protein [Erwinia phage pEa_SNUABM_29]		
**54**	33898-34329	hypothetical protein [Erwinia phage vB_EamM_Stratton]		
**55**	34316-34702	hypothetical protein ASESINO_151 [Erwinia phage vB_EamM_Asesino]		
**56**	34844-35290	hypothetical protein [Enterobacteria phage SEGD1]		
**57**	35352-35570	hypothetical protein [Salmonella phage SPFM4]		
**58**	35751-36548	virion structural protein [Erwinia phage phiEaH2]		
**59**	36559-37401	virion structural protein [Salmonella phage SPN3US]		
**60**	37412-38257	virion structural protein [Erwinia phage vB_EamM_Huxley]		
**61**	38279-39190	virion structural protein [Salmonella phage SPLA1a]		
**62**	39260-40099	virion structural protein [Erwinia phage vB_EamM_ChrisDB]		
**63**	40110-40997	virion structural protein [Erwinia phage vB_EamM_Caitlin]		
**64**	41001-41828	virion structural protein [Erwinia phage vB_EamM_Kwan]		
**65**	41876-43288	virion structural protein [Salmonella phage SPLA5c]		
**66**	43300-44298	glycosyl hydrolase [Proteus phage 7]		
**67**	44308-45231	glycosyl hydrolase [Proteus phage 7]		
**68**	45245-46663	virion structural protein [Salmonella phage SPLA3]		
**69**	46676-47665	glycosyl hydrolase [Salmonella phage MET_P1_082_240]		
**70**	47668--48567	virion structural protein [Enterobacteria phage SEGD1]		
**71**	48659-48823	virion structural protein [Salmonella phage SPLA1a]		

ORF	Gene Positions	Functions	Name	Accession
**72**	49060-50317	Phage tail fibre adhesin Gp38	GP38 superfamily	cl05026
**73**	50285-50902	endolysin [Salmonella phage SPN3US]	Lysozyme family protein	cl34694
**74**	51191-51832	tail assembly chaperone [Erwinia phage phiEaH2]		
**75**	51842-52357	putative peptidase M15A [Erwinia phage vB_EamM_Phobos]		
**76**	52483-52905	hypothetical protein [Escherichia phage vB_EcoM_Lh1B]		
**77**	53087-53758	hypothetical protein [Erwinia phage vB_EamM_Parshik]		
**78**	53734-54300	hypothetical protein [Salmonella phage JN03]		
**79**	54263-55591	putative radical SAM superfamily protein 1 [Salmonella phage STsAS]	rSAM_Cxxx_rpt superfamily	cl33264
**80**	55584-56498	radical SAM domain-containing protein [Salmonella phage SPLA5c]	Radical SAM superfamily	cl18962
**81**	56520-57407	hypothetical protein [Salmonella phage SPLA3]		
**82**	57648-57911	hypothetical protein [Salmonella phage SaP7]		
**83**	58525-59727	virion structural protein [Erwinia phage phiEaH2]	Smc superfamily	cl34174
**84**	59775-64958	hypothetical protein [Salmonella phage JN03]		
**85**	65054-69184	virion structural protein [Salmonella phage SPLA5c]		
**86**	69197-72901	hypothetical protein [Escherichia phage vB_EcoM_EC001]		
**87**	72969-74231	hypothetical protein [Escherichia phage vB_EcoM_Lh1B]		
**88**	74293-74502	hypothetical protein [Enterobacteria phage SEGD1]		
**89**	74710-75642	baseplate assembly protein [Erwinia phage vB_EamM_ChrisDB]		
**90**	75827-76144	hypothetical protein [Escherichia phage vB_EcoM_EC001]		
**91**	76265-77503	hypothetical protein ACEC001_1750 [Escherichia phage vB_EcoM_EC001]		
**92**	77564-78145	hypothetical protein [Salmonella phage JN03]		
**93**	78155-78718	hypothetical protein SPLA5c_PHROGS00183 [Salmonella phage SPLA5c]		
**94**	78718-79245	hypothetical protein [Salmonella phage SPN3US]		
**95**	79325-79912	hypothetical protein [Escherichia phage vB_EcoM_EC001]		

ORF	Gene Positions	Functions	Name	Accession
**96**	80018-80419	hypothetical protein [Salmonella phage JN03]		
**97**	80406-80897	hypothetical protein [Escherichia phage vB_EcoM_EC001]		
**98**	80920-81330	hypothetical protein SEGD1_186 [Enterobacteria phage SEGD1]		
**99**	81344-81727	hypothetical protein [Escherichia phage vB_EcoM_Lh1B]		
**100**	81857-82357	hypothetical protein [Salmonella phage SPFM4]		
**101**	82412-83038	hypothetical protein [Erwinia phage phiEaH2]		
**102**	83135-85645	putative SMC domain-containing protein [Escherichia phage vB_EcoM_EC001]	PRK01156 superfamily	cl30905
**103**	85823-85927	hypothetical protein [Enterobacteria phage SEGD1]		
**104**	85914-86117	hypothetical protein [Salmonella phage SPN3US]		
**105**	86101-86232	hypothetical protein [Salmonella phage SPFM4]		
**106**	86771-87259	acetyltransferase [Salmonella phage SPLA1a]		
**107**	87331-87540	putative elongation factor Tu [Erwinia phage vB_EamM_ChrisDB]		
**108**	87537-88055	hypothetical protein [Erwinia phage pEa_SNUABM_6]		
**109**	88074-88496	hypothetical protein [Salmonella phage SPLA5c]		
**110**	88497-88718	hypothetical protein [Escherichia phage vB_EcoM_EC001]		
**111**	88696-89196	putative Appr-1-p processing protein [Erwinia phage vB_EamM_Stratton]	Macro_SF superfamily	cl00019
**112**	89296-90018	endolysin [Enterobacteria phage SEGD1]	Lyz-like superfamily	cl00222
**113**	90015-90608	hypothetical protein [Erwinia phage pEa_SNUABM_29]		
**114**	90987-92120	hypothetical protein [Escherichia phage vB_EcoM_EC001]		
**115**	92180-92803	hypothetical protein [Erwinia phage pEa_SNUABM_29]	HMMR_N superfamily	cl25727
**116**	92814-94193	virion structural protein [Salmonella phage SPLA5c]		
**117**	94218-94727	hypothetical protein [Salmonella phage SPLA3]		
**118**	94737-95216	hypothetical protein [Erwinia phage pEa_SNUABM_6]	Flavin_utilizing_monoxygenases	cl19096
**119**	95304-96023	putative endodeoxyribonuclease [Salmonella phage JN03]	DnaQ_like_exo superfamily	cl10012

ORF	Gene Positions	Functions	Name	Accession
**120**	96023-97537	ribonuclease [Salmonella phage SPLA3]	RNase_H_like superfamily	cl14782
**121**	97539-98279	hypothetical protein [Salmonella phage SPN3US]		
**122**	98333-99067	hypothetical protein [Escherichia phage vB_EcoM_EC001]		
**123**	99081-99452	hypothetical protein SEGD1_214 [Enterobacteria phage SEGD1]		
**124**	99463-99870	hypothetical protein [Salmonella phage SPFM14]		
**125**	99881-100420	hypothetical protein [Erwinia phage pEa_SNUABM_29]		
**126**	100417-100959	hypothetical protein [Escherichia phage vB_EcoM_EC001]		
**127**	101028-101951	hypothetical protein [Salmonella phage SaP7]		
**128**	101999-102754	hypothetical protein [Salmonella phage STsAS]		
**129**	102853-103152	hypothetical protein [Erwinia phage pEa_SNUABM_29]		
**130**	103168-104661	putative recombinase [Erwinia phage pEa_SNUABM_8]		
**131**	104707-104982	hypothetical protein [Erwinia phage vB_EamM_ChrisDB]		
**132**	104979-105647	RNA polymerase beta subunit [Erwinia phage vB_EamM_Asesino]		
**133**	105644-106369	putative virion structural protein [Erwinia phage vB_EamM_Asesino]		
**134**	106320-106787	hypothetical protein [Escherichia phage vB_EcoM_Lh1B]		
**135**	106864-107394	hypothetical protein [Salmonella phage SPLA5c]		
**136**	107428-108306	hypothetical protein [Escherichia phage vB_EcoM_EC001]		
**137**	108287-109561	hypothetical protein [Enterobacteria phage SEGD1]		
**138**	109571-110422	hypothetical protein [Erwinia phage vB_EamM_Stratton]		
**139**	110409-111068	hypothetical protein [Salmonella phage SPLA3]		
**140**	111127-111489	hypothetical protein [Salmonella phage SaP7]		
**141**	111538-111957	hypothetical protein [Erwinia phage phiEaH2]		
**142**	111950-112810	hypothetical protein [Salmonella phage SPAsTU]		
**143**	112810-113841	thymidylate synthase [Escherichia phage vB_EcoM_EC001]	thymidylate synthase	cl19097

ORF	Gene Positions	Functions	Name	Accession
**144**	113854-114390	hypothetical protein [Proteus phage 7]		
**145**	114387-114638	hypothetical protein [Salmonella phage STsAS]		
**146**	114640-115407	hypothetical protein [Salmonella phage JN03]		
**147**	115419-115898	hypothetical protein [Escherichia phage vB_EcoM_Lh1B]		
**148**	115911-116099	hypothetical protein [Erwinia phage pEa_SNUABM_29]		
**149**	116164-117105	nucleotide pyrophosphohydrolase [Salmonella phage SPLA1a]	NTP-PPase superfamily	cl16941
**150**	117080-117502	hypothetical protein [Salmonella phage JN03]		
**151**	117558-118109	virion structural protein [Erwinia phage vB_EamM_Asesino]		
**152**	118132-120354	putative virion structural protein [Salmonella phage SPAsTU]		
**153**	120403-127524	lytic transglycosylase [Proteus phage 7]	Lyz-like superfamily	cl00222
**154**	127554-129167	RNA polymerase beta subunit [Erwinia phage vB_EamM_Asesino]		
**155**	129186-133391	phage DNA-directed RNA polymerase beta subunit 2 [Salmonella phage SPFM4]		
**156**	133439-133732	hypothetical protein [Erwinia phage vB_EamM_ChrisDB]		
**157**	133729-135204	hypothetical protein [Salmonella phage MET_P1_082_240]		
**158**	135220-135942	virion structural protein [Erwinia phage phiEaH2]		
**159**	136005-136808	head maturation protease [Erwinia phage phiEaH2]	Peptidase_S80	pfam20034
**160**	136812-137441	hypothetical protein [Erwinia phage vB_EamM_ChrisDB]	Peptidase_S80	pfam20034
**161**	137497-137796	hypothetical protein [Escherichia phage vB_EcoM_Lh1B]		
**162**	137850-138398	putative DNA repair exonuclease [Vibrio phage vB_pir03]	HAD_like superfamily	cl21460
**163**	138545-138943	hypothetical protein [Salmonella phage JN03]		
**164**	138998-139387	hypothetical protein [Erwinia phage pEa_SNUABM_29]		
**165**	139422-140093	putative phage HD domain protein [Salmonella phage SPFM4]		
**166**	140120-140608	hypothetical protein SEGD1_257 [Enterobacteria phage SEGD1]		
**167**	140608140970	hypothetical protein [Salmonella phage SPFM9]		

ORF	Gene Positions	Functions	Name	Accession
**168**	141027-141569	hypothetical protein [Salmonella phage SPLA3]		
**169**	141619-142494	putative major virion structural protein [Bacillus phage vB_BspM_AgentSmith]		
**170**	142498-144543	putative tail sheath protein [Escherichia phage vB_EcoM_EC001]		
**171**	144601-145506	virion structural protein [Klebsiella phage vB_KvM-Eowyn]		
**172**	145517-148030	putative virion structural protein [Salmonella phage SPAsTU]		
**173**	148033-149676	virion structural protein [Erwinia phage phiEaH2]		
**174**	149721-151832	terminase large subunit [Salmonella phage pSal-SNUABM-04]	large terminase protein	cl28557
**175**	151832-152071	hypothetical protein ACQ60_gp005 [Salmonella phage SPN3US]		
**176**	152138-153514	putative virion structural protein [Salmonella phage SPFM4]		
**177**	153587-154720	hypothetical protein [Salmonella phage JN03]		
**178**	154731-155240	hypothetical protein [Proteus phage 7]		
**179**	157011-157586	hypothetical protein [Salmonella phage JN03]		
**180**	157583-158113	putative ATP-dependent protease, HslV subunit [Erwinia phage pEa_SNUABM_43]	Ntn_hydrolase superfamily	cl00467
**181**	158318-158560	helicase superfamily 1/2, ATP-binding protein [Serratia phage vB_SmaM-Sureiya]		
**182**	159347-159583	hypothetical protein [Salmonella phage SaP7]		
**183**	159862-160683	hypothetical protein [Salmonella phage SPLA3]	cond_enzymes superfamily	cl09938
**184**	160908-161858	hypothetical protein [Salmonella phage SPLA1a]		
**185**	161941-162257	hypothetical protein [Salmonella phage SPFM4]		
**186**	162425-162814	hypothetical protein [Erwinia phage pEa_SNUABM_29]		
**187**	162866-163678	hypothetical protein [Pseudomonas phage 201phi2-1]		
**188**	163842-164162	hypothetical protein [Erwinia phage vB_EamM_Stratton]		
**189**	164159-164722	hypothetical protein [Salmonella phage MET_P1_082_240]		
**190**	165019-165426	hypothetical protein [Enterobacteria phage SEGD1]		
**191**	165419-165928	hypothetical protein [Escherichia phage vB_EcoM_Lh1B]		

ORF	Gene Positions	Functions	Name		Accession
**192**	165925-166413	hypothetical protein [Salmonella phage SPAsTU]			
**193**	166410-166841	hypothetical protein [Salmonella phage SPN3US]			
**194**	166851-167450	hypothetical protein [Escherichia phage vB_EcoM_EC001]			
**195**	167450-167860	hypothetical protein [Erwinia phage pEa_SNUABM_9]			
**196**	167951-170242	DNA polymerase [Salmonella phage SPLA5c]			
**197**	170356-170628	hypothetical protein [Salmonella phage SaP7]			
**198**	170615-170812	hypothetical protein [Salmonella phage SPLA3]			
**199**	170896-171963	hypothetical protein [Salmonella phage STsAS]			
**200**	172170-174023	hypothetical protein pEaSNUABM6_00039 [Erwinia phage pEa_SNUABM_6]			
**201**	174106-175614	DNA-directed RNA polymerase alpha subunit [Proteus phage 7]			
**202**	175818-176057	RNA polymerase beta subunit [Salmonella phage SPLA1a]			
**203**	176071-176652	hypothetical protein [Erwinia phage vB_EamM_Stratton]			
**204**	176734-177120	hypothetical protein [Erwinia phage vB_EamM_Asesino]			
**205**	177120-177794	hypothetical protein [Salmonella phage SPLA3]			
**206**	177778-178926	SbcD-like subunit of palindrome specific endonuclease [Salmonella phage SPLA1a]	SbcD superfamily		cl33866
**207**	178923-179759	hypothetical protein [Erwinia phage pEa_SNUABM_29]			
**208**	179828-180484	hypothetical protein [Salmonella phage SPN3US]			
**209**	180620-182254	hypothetical protein [Erwinia phage phiEaH2]			
**210**	182267-183967	hypothetical protein [Erwinia phage pEa_SNUABM_29]			
**211**	183980-185347	hypothetical protein [Escherichia phage vB_EcoM_Lh1B]			
**212**	185385-185708	hypothetical protein [Erwinia phage vB_EamM_Asesino]			
**213**	185775-188012	putative DNA-directed RNA polymerase beta subunit 2 [Salmonella phage STsAS]			
**214**	188012-190117	DNA-directed RNA polymerase alpha subunit [Salmonella phage MET_P1_082_240]			
**215**	190164-192908	terminase large subunit [Salmonella phage SPLA3]	Hop superfamily	NC	cl34242

ORF	Gene Positions	Functions	Name	Accession
**216**	193240-193860	hypothetical protein [Salmonella phage SaP7]		
**217**	193853-195823	hypothetical protein [Salmonella phage MET_P1_082_240]		
**218**	195868-196239	hypothetical protein SEGD1_042 [Enterobacteria phage SEGD1]		
**219**	196321-197178	hypothetical protein [Salmonella phage JN03]		
**220**	197165-198460	DNA-directed RNA polymerase, beta subunit [Escherichia phage vB_EcoM_EC001]		
**221**	198474-198974	hypothetical protein [Escherichia phage vB_EcoM_Lh1B]		
**222**	199026-200810	DNA polymerase [Salmonella phage SPLA1a]		
**223**	200881-202233	beta-barrel assembly-enhancing protease [Salmonella phage MET_P1_082_240]		
**224**	202249-202692	putative DNA polymerase family B [Erwinia phage pEa_SNUABM_8]		
**225**	202689-204422	hypothetical protein ACEC001_0500 [Escherichia phage vB_EcoM_EC001]		
**226**	204465-207425	putative virion structural protein 2 [Salmonella phage JN03]		
**227**	207425-208684	putative virion structural protein 3 [Salmonella phage STsAS]		
**228**	208710-209828	putative virion structural protein [Escherichia phage vB_EcoM_Lh1B]		
**229**	209839-210771	putative virion structural protein [Enterobacteria phage SEGD1]		
**230**	210795-211352	hypothetical protein [Salmonella phage STsAS]		
**231**	211409-212686	internal head protein [Erwinia phage vB_EamM_ChrisDB]		
**232**	212764-214023	internal head protein [Erwinia phage vB_EamM_Asesino]		
**233**	214168-214923	hypothetical protein [Salmonella phage JN03]		
**234**	214981-215550	hypothetical protein [Salmonella phage SPAsTU]		
**235**	215597-215977	hypothetical protein [Salmonella phage pSal-SNUABM-04]		
**236**	215974-216495	hypothetical protein [Erwinia phage pEa_SNUABM_6]		
**237**	216492-217043	putative dihydrofolate reductase [Enterobacteria phage SEGD1]	FolA	COG0262
**238**	217045-217374	hypothetical protein [Salmonella phage MET_P1_082_240]		

ORF	Gene Positions	Functions	Name	Accession
**239**	217402-217992	hypothetical protein STsAS_043 [Salmonella phage STsAS]		
**240**	218068-219570	hypothetical protein SEGD1_062 [Enterobacteria phage SEGD1]		
**241**	219545-220900	putative virion structural protein [Escherichia phage vB_EcoM_EC001]		
**242**	220903-221442	hypothetical protein [Escherichia phage vB_EcoM_Lh1B]		
**243**	221442-222755	putative virion structural protein [Enterobacteria phage SEGD1]		
**244**	222799-223596	hypothetical protein [Escherichia phage vB_EcoM_Lh1B]		
**245**	223839-223982	hypothetical protein [Salmonella phage MET_P1_082_240]		
**246**	223982-224164	hypothetical protein STsAS_050 [Salmonella phage STsAS]		
**247**	224164-224352	hypothetical protein SEGD1_069 [Enterobacteria phage SEGD1]		
**248**	224412-224828	hypothetical protein SPFM8_00217 [Salmonella phage SPFM8]		
**249**	224831-225691	hypothetical protein [Enterobacteria phage SEGD1]		
**250**	225805-225951	hypothetical protein ACQ60_gp195 [Salmonella phage SPN3US]		
**251**	226064-226210	hypothetical protein [Salmonella phage MET_P1_082_240]		
**252**	226345-227907	DnaB-like replicative helicase [Erwinia phage phiEaH2]		
**253**	227922-228446	hypothetical protein [Escherichia phage vB_EcoM_Lh1B]		
**254**	228459-230759	putative major capsid protein [Enterobacteria phage SEGD1]		
**255**	230893-231090	hypothetical protein [Salmonella phage SPAsTU]		
**256**	231149-132153	hypothetical protein [Escherichia phage vB_EcoM_Lh1B]		
**257**	232153-233943	polymerase [Erwinia phage vB_EamM_Asesino]		
**258**	233975-235645	hypothetical protein [Enterobacteria phage SEGD1]		
**259**	235711-235977	hypothetical protein [Salmonella phage SPFM12]		
**260**	236012-236314	hypothetical protein [Proteus phage 7]		

ORF	Gene Positions	Functions	Name	Accession
**261**	236378-239122	virion structural protein [Escherichia phage vB_EcoM_EC001]		
**262**	239134-240651	virion structural protein [Enterobacteria phage SEGD1]		
